# Supplementary material for: Antibacterial Activity and Mechanism of GO/Cu2O/ZnO Coating on Ultrafine Glass Fiber
Source: Nanomaterials (Basel). 2022 May 29;12(11):1857. doi: 10.3390/nano12111857 (PMC9181844; doi:10.3390/nano12111857)
Supplement: Supplementary file 1 [file nanomaterials-12-01857-s001.zip › nanomaterials-1738779-supplementary.pdf]

Supporting Information

Antibacterial Activity and Mechanism of GO/Cu_2O/ZnO Coating on Ultrafine Glass Fiber

Manna Li [1,2,3], Zhaofeng Chen [1,*], Lixia Yang [1,*], Jiayu Li [1], Jiang Xu [1], Chao Chen [1,2,3], Qiong Wu [1,2], Mengmeng Yang [1,2] and Tianlong Liu [1,2]

1 International Laboratory for Insulation and Energy Efficiency Materials, College of Materials Science and Technology, Nanjing University of Aeronautics and Astronautics, Nanjing 211106, China; nuaa_lmn@163.com (M.L.); ljy0822@mail.nwpu.edu.cn (J.L.); xujiang73@nuaa.edu.cn (J.X.); chenchaojsw@163.com (C.C.); wuqiong@nuaa.edu.cn (Q.W.); yangmengmeng@nuaa.edu.cn (M.Y.); liu1061178707@nuaa.edu.cn (T.L.)

2 Jiangsu Collaborative Innovation Center for Advanced Inorganic Function Composites, Nanjing University of Aeronautics and Astronautics, Nanjing 211106, China

3 Suqian Kongtian New Materials Co., Ltd., Suqian 223800, China

* Correspondence: zhaofeng_chen@163.com (Z.C.); lixiayang@nuaa.edu.cn (L.Y.); Tel.: +86-18952018969 (Z.C.); Tel.: +86-19850822455 (L.Y.)

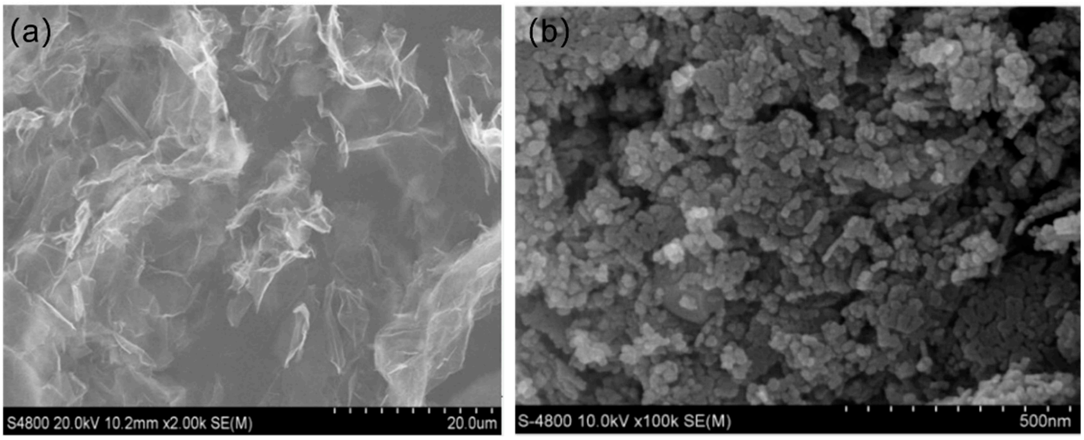

Figure S1 SEM picture. (a) GO nanosheets SEM picture, (b) ZnO nanopartical.
